# Optimized ^31^P MRS in the human brain at 7 T with a dedicated RF coil setup

**DOI:** 10.1002/nbm.3422

**Published:** 2015-10-07

**Authors:** Bart L. van de Bank, Stephan Orzada, Frits Smits, Miriam W. Lagemaat, Christopher T. Rodgers, Andreas K. Bitz, Tom W. J. Scheenen

**Affiliations:** ^1^ Department of Radiology and Nuclear MedicineRadboud University Medical CenterNijmegenThe Netherlands; ^2^ Erwin L. Hahn InstituteUniversity Hospital Duisburg‐EssenEssenGermany; ^3^ Oxford Centre for Clinical Magnetic Resonance Research (OCMR)University of Oxford, John Radcliffe HospitalOxfordUK; ^4^ Medical Physics in RadiologyGerman Cancer Research Center (DKFZ)HeidelbergGermany

**Keywords:** ^31^P‐MRS, ^31^P‐MRSI, spectroscopic imaging, ultra‐high field, RF coil, array coil, multi‐transmit, *B*_1_ shimming, WSVD, 7 T

## Abstract

The design and construction of a dedicated RF coil setup for human brain imaging (^1^H) and spectroscopy (^31^P) at ultra‐high magnetic field strength (7 T) is presented. The setup is optimized for signal handling at the resonance frequencies for ^1^H (297.2 MHz) and ^31^P (120.3 MHz). It consists of an eight‐channel ^1^H transmit–receive head coil with multi‐transmit capabilities, and an insertable, actively detunable ^31^P birdcage (transmit–receive and transmit only), which can be combined with a seven‐channel receive‐only ^31^P array. The setup enables anatomical imaging and ^31^P studies without removal of the coil or the patient. By separating transmit and receive channels and by optimized addition of array signals with whitened singular value decomposition we can obtain a sevenfold increase in SNR of ^31^P signals in the occipital lobe of the human brain compared with the birdcage alone. These signals can be further enhanced by 30 ± 9% using the nuclear Overhauser effect by *B*_1_‐shimmed low‐power irradiation of water protons. Together, these features enable acquisition of ^31^P MRSI at high spatial resolutions (3.0 cm^3^ voxel) in the occipital lobe of the human brain in clinically acceptable scan times (~15 min). © 2015 The Authors. NMR in Biomedicine published by John Wiley & Sons Ltd.

**Abbreviations used**
*|E|*: *absolute component of electric field*
*|H|*: *absolute component of magnetic field*
*^1^H*: *proton*
*^31^P*: *phosphorus‐31*
*ATP*: *adenosine triphosphate*
*BC*: *birdcage*
*CH*: *channel*
*CP mode*: *circularly polarized mode (quadrature driven)*
*FOV*: *field of view*
*GPC*: *glycerophosphocholine*
*GPE*: *glycerophosphoethanolamine*
*NOE*: *nuclear Overhauser effect*
*PC*: *phosphocholine*
*PCr*: *phosphocreatine*
*PE*: *phosphoethanolamine*
*P_i_*: *inorganic phosphate*
*Q_L_*: *quality factor in loaded condition*
*Q_U_*: *quality factor in unloaded condition*
*ROI*: *region of interest*
*Rx*: *signal reception*
*S_12_*: *transmission coefficient*
*SAR*: *specific absorption rate*
*SNR*: *signal‐to‐noise ratio*
*T_acq_*: *total acquisition time*
*TEM*: *transverse electromagnetic*
*Tx*: *signal transmission*
*VNA*: *vector network analyzer*
*WSVD*: *whitened singular value decomposition*.

## Introduction

For many years now, examining the local distribution of metabolites containing phosphorus (^31^P) atoms in the human brain has been done non‐invasively by *in vivo* ^31^P‐MRSI 1. This can give insight in determining or understanding specific metabolic processes, such as energy metabolism or cell membrane turnover. Metabolites of interest for these processes are phosphocreatine (PCr), adenosine triphosphate (ATP), inorganic phosphate (P_i_) and the phosphomono‐ and diesters, which can be directly observed *in vivo* by ^31^P spectroscopy.

The sensitivity of the ^31^P nucleus is lower than that of the ^1^H nucleus (*γ* ^1^H/*γ* ^31^P = 2.4), but both improve with increasing *B*_0_. Moreover, ^1^H *B*_1_ homogeneity becomes a real challenge in larger volumes at higher field strengths (≥7 T), but this is less so for ^31^P. Rodgers *et al* found a 2.8‐fold increase for ^31^P‐MRS in the human heart when going from 3 T to 7 T 2. The complexity of spectral patterns of ^31^P‐MRS is reduced compared with ^1^H spectra, because fewer metabolites have a ^31^P nucleus present, and fewer resonances overlap.

Dedicated hardware is needed to detect MR signals from ^31^P nuclei. A clever design of this hardware can contribute to an improved signal‐to‐noise ratio (SNR) for detection of these signals. RF surface coils, regardless of their operating frequency, have been frequently used for their excellent, albeit spatially constrained, SNR 3. A drawback of RF surface coils is their inhomogeneous *B*_1_ field, requiring complex high‐power adiabatic pulses to homogeneously excite a region of interest (ROI) close to the coil 4. Instead, a fairly uniform flip can be achieved by using volume coils, of course depending on the size of the sample and the operating frequency of the coil. This enables the use of short hard pulses instead of longer adiabatic pulses, as has been shown by Avdievich and Hetherington at 4 T 5. When separating the excitation and reception of MR signals by designing a volume RF coil for excitation and a receive array for acquisition, adiabatic pulse shapes can be avoided and excellent SNR can still be achieved by receiving with small elements 2, 3, 6, 7. The signals from each of these elements must then be combined optimally, which can be achieved with the whitened singular value decomposition (WSVD) method 8, 9.

The ^31^P RF coil setup needs to be integrated into another RF coil that handles ^1^H signals. In contrast to clinical MR systems (at *B*_0_ ≤ 3 T), no whole body RF coil is provided as standard within human 7 T systems 10. Therefore, local ^1^H RF coils must be employed to excite the proton spins and receive their signals, to obtain anatomical images and to be able to optimize the field (*B*_0_ and *B*_1_ shimming). The short wavelengths produce inhomogeneous RF fields; these destructive interferences can coincide within an ROI, decreasing signal intensity at the desired location. When using multiple signal transmission (Tx) coil elements, it is possible to adjust the transmitted RF field by changing input phases and amplitudes of the RF pulses to remove (or shift) these interferences outside the ROI, known as *B*_1_ shimming 11. When multiple coil elements are integrated within a multi‐transmit proton coil it must be assured that local specific absorption rate (SAR) hot‐spots are minimized and remain within guidelines. If by *B*_1_ shimming proton signals of water can be irradiated with low RF power, the nuclear Overhauser effect (NOE) 12 can be used to increase the steady state magnetization of certain ^31^P nuclei surrounded by water, potentially providing an additional sensitivity to a ^31^P experiment. This enhancement technique can be used in any organ as long as sufficient power to excite the ^1^H spins can be delivered to the ROI. Positive NOE enhancements have been reported for prostate and brain ^31^P MRSI at 7 T 13, 14. Other techniques, such as INEPT, can also be used to enhance signals of the ^31^P compounds, as has been shown for 3 T 15, 16 and even for 7 T 17, 18. However, as these techniques use rather complex sequences with several inversion pulses, reaching the correct flip angle and not running into SAR restrictions is a major issue. Moreover, *T*_2_ relaxation during long spin evolution times at 7 T could decrease or even negate the polarization gain.

Our aim was to design and construct an integrated coil setup optimized for efficient NOE, uniform ^31^P spin excitation and high SNR ^31^P MRS(I) for 7 T. This safety‐validated coil setup should allow optimization of the ^1^H transmit field (*B*_1_ shimming) and standard ^1^H imaging as well as uniform excitation and acquisition of ^31^P spins throughout the human brain, with a possibility of using an additional local receive array and without removing the coil or the patient during an examination. Therefore, we combined an eight‐channel (8‐CH) multi‐transmit head coil (^1^H) with a newly constructed and insertable actively detunable volume resonator (^31^P), and developed a 7‐CH receive‐only (Rx) array (^31^P) that improves SNR locally. Together with optimized signal addition and the possibility to use *B*_1_‐shimmed NOE the sensitivity of local ^31^P signal detection is maximized.

## Materials and Methods

### RF coil design and construction

The complete coil setup consists of three parts: an 8‐CH ^1^H transceiver array (TxRx), an actively detunable ^31^P volume resonator and a ^31^P 7‐CH Rx array. Note that the ^31^P volume resonator can be used as transceiver (without Rx array) and as transmit only (with Rx array).

#### ^1^H TxRx array

As basis for the complete setup we chose to use an existing ^1^H head coil designed by Orzada *et al*. 19. This coil has an octagonal shaped construction and consists of eight intrinsically decoupled microstrip elements with meanders that can be used in multi‐transmit mode 20. Each element was tuned to the resonance frequency of 297.2 MHz and matched to 50 Ω when loaded and with the birdcage (BC) inserted. Cable traps tuned to the resonance frequency of ^31^P were added to all individual elements.

#### ^31^P volume resonator (TxRx)

**Figure 1:**
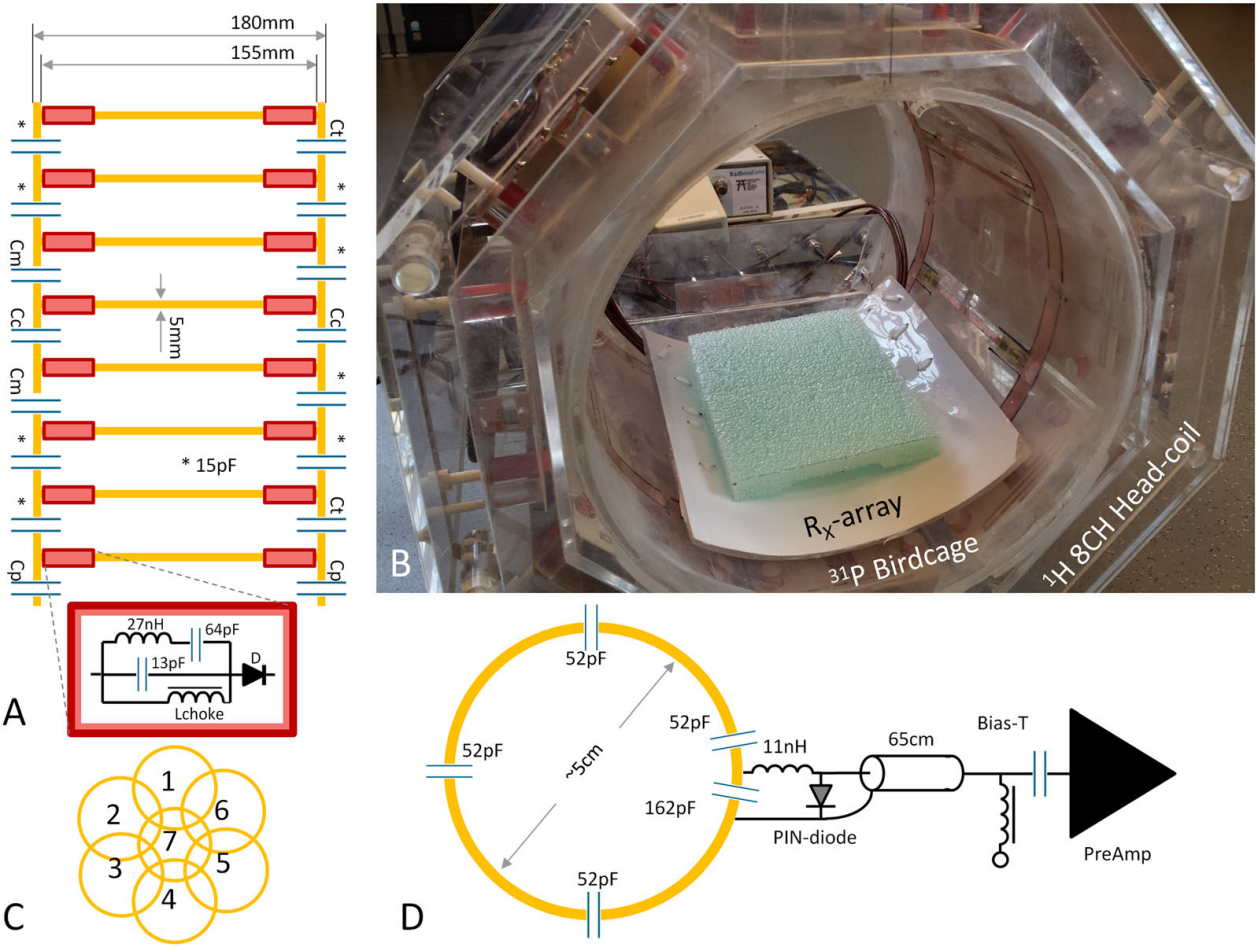
Overview of the complete setup, with detailed representations of the newly designed, detunable ^31^P BC insert and ^31^P Rx array. (A) Schematic representation of the eight‐rung high‐pass BC coil; included are details of the tank and detune circuits (red box). Capacitor values used to tune and match the BC coil were *C*_m_ = 15.6 pF, *C*_t_ = 13.3 pF, *C*_c_ = 27 pF and *C*_p_ = 10 pF. (B) The octagonal‐shaped 8‐CH ^1^H head coil was used as the basis for the insertable, detunable ^31^P BC coil, which could host the additional 7‐CH Rx array coil. (C) Schematic configuration of the 7‐CH Rx array. (D) A pictorial overview of a single element of the 7‐CH Rx array, where each adjacent element is decoupled by overlap and by pre‐amplifier decoupling.

The ^31^P coil was designed as a BC coil to allow uniform excitation of the ^31^P signals in the human brain, which should fit inside the 8‐CH head coil. Therefore, we created an eight‐rung, high‐pass design. This design avoids interference of the resonant modes with the 8‐CH head coil, as the highest useful resonance is the dominant mode 21. This eight‐rung design also ensures that the rungs could be positioned exactly between the eight ^1^H microstrip elements (Fig. 1B).

**Figure 2:**
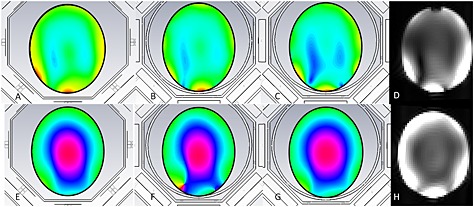
(A–C, E–G) Simulation of proton magnetic field (A–C) and phase (E–G) distribution of the 8‐CH head coil, with and without the ^31^P BC inserted into the 8‐CH setup. (A, E) The field and phase distributions of the 8‐CH head only, (B, F) the distribution when the BC is inserted (in this case the legs had a width of 12.5 mm), (C, G) the distribution when the width of the legs is reduced to 4.5 mm. (D, H) ^1^H images of the same phantom with the BC present in the setup; the image in D corresponds to the simulation result as presented in C, hence showing equal field distribution. In H the field distribution is shown with the tank circuits inserted into the BC.

The BC was created with copper foil (Parker Chomerics CHO‐FOIL®, Parker Hannifin Corporation, Woburn, MA, USA) attached to a Plexiglas tube (*Ø*_out_ 24 cm, length 25 cm, thickness 0.5 cm). We enlarged the diameter to 25 cm by opening the tube at one side over the complete length. The opening was fixed by two small Plexiglass parts (*l* × *w* 3.2 × 3.2 cm^2^), which supported the end rings. Two rings of copper foil (width 12.5 mm) were attached to the tube with a spacing of 155 mm. Between the end rings, a total of eight rungs (length 15.5 cm, width 4.5 mm) were placed equidistantly around the tube. A smaller width of the rungs compared with the end rings was chosen to minimize distortion of the ^1^H field (Fig. 2). The BC was tuned to 120.3 MHz (loaded), by soldering a total capacitance of 14.7 pF in the end rings between the rungs, where a capacitance of 16 pF had been determined with BirdcageBuilder 22. Two tank circuits were added to each rung. Symmetry was preserved by positioning them at both ends. These circuits cause a high impedance at the frequency of 297.2 MHz, while a low impedance is present at 120.3 MHz, hence minimizing the coupling to the ^1^H elements and optimizing tuning to the ^31^P Larmor frequency 23. Additionally, a PIN‐diode was placed in series (Fig. 1A) to actively detune the BC during signal acquisition and to enable the use of a local receive array coil. The BC coil had two ports and was driven in circularly polarized (CP) mode (quadrature mode, fixed phase difference of 90° between the two ports). Each port was matched to the characteristic impedance of the MR system (50 Ω) by increasing the matching capacitors to 15.6 pF and by decreasing the capacitors in the opposing end ring to 13.9 pF. The ports were isolated by replacing the capacitors between the matching circuits and the other end ring by 27 pF and by reducing the values of both capacitors that were in the same plane to 8.6 pF. Cable traps tuned to 120.3 MHz to balance the input and to filter common‐mode currents were inserted between the matching circuit and the connection to the MR system.

#### Array coil (Rx only)

A 7‐CH Rx array coil was developed to further improve the SNR of signals arising from ^31^P compounds. Each circular loop (channel) was made from copper wire (wire diameter 1.25 mm^2^), having a loop radius of approximately 25 mm and having four symmetrically distributed fixed tuning capacitors (68 pF each). Each loop was matched to 50 Ω (loaded) by placing an additional capacitor (162 pF), which was also part of the active detuning network, in the loop. Part of this active detuning is the parallel positioned PIN‐diode. To create a high impedance at the resonance frequency in the loop, a small inductor of 11 nH was added between the PIN‐diode and the matching capacitor (Fig. 1D). Mutual inductance was minimized by overlapping adjacent loops. Coupling between non‐overlapping loops was reduced by pre‐amplifier decoupling, which transformed the low impedance of the pre‐amplifier to a high impedance at the coil 6, 7.

### MR hardware and RF interfaces

All experiments were performed on a 7 T whole body MR system (MAGNETOM 7 T, Siemens Healthcare, Erlangen, Germany). The 8‐CH ^1^H coil was driven by eight 1 kW RF amplifiers (LPPA 13080 W, Dressler, Germany), where the amplitude and phase of each channel could be altered using a vector modulator, enabling *B*_1_ shimming 11. The TxRx switches with pre‐amps were placed in a separate box at the head of the patient table.

A single 8 kW RF amplifier was used to excite the ^31^P signal (LPPA 13080 W‐CAN, Dressler, Germany). This signal was divided and phased by a lumped‐element quad‐hybrid to drive the BC in quadrature mode. The ^31^P receive array was connected to a homebuilt 7‐CH receive interface box optimized for signal acquisition at 120.3 MHz, where in each receive‐path an improved tank circuit 23 was added to trap proton signals. For safety we added to the interface a PIN‐diode breakdown detection circuit, that immediately stops the scanner if a PIN‐diode malfunctions or when a cable is disconnected.

### Functionality and safety tests

#### Bench tests

The BC was tested in TxRx mode and in Tx mode. In the latter case, the coil must be detuned while receiving the MR signal with the local Rx setup. Active tuning was verified by measuring the transmission coefficient (*S*_12_) of the BC, by using two loosely coupled pick‐up probes that were connected to a vector network analyzer (VNA) (R&HZVL3, Rohde & Schwarz, Munich, Germany). This setup was also used to determine the ratio of unloaded‐to‐loaded quality factors (*Q*_U_/*Q*_L_) 24.

For the receive array, we assessed active detuning, determined the crosstalk between each element, and determined the ratio of unloaded‐to‐loaded quality factors (*Q*_U_/*Q*_L_) for each element. Active detuning was checked by switching the currents to the PIN‐diode between forward and reverse bias, while measuring the effect of the *S*_12_ response of two pick‐up loops that were weakly coupled to the element under investigation. This method was also used to determine the *Q*‐factor. Crosstalk between elements was determined by connecting the elements under investigation to the network analyzer, probing signal through one of the elements and receiving with the other. Note that only the element under investigation was tuned and all other coil elements were detuned while making these measurements.

#### Simulations and RF field measurements

**Figure 3:**
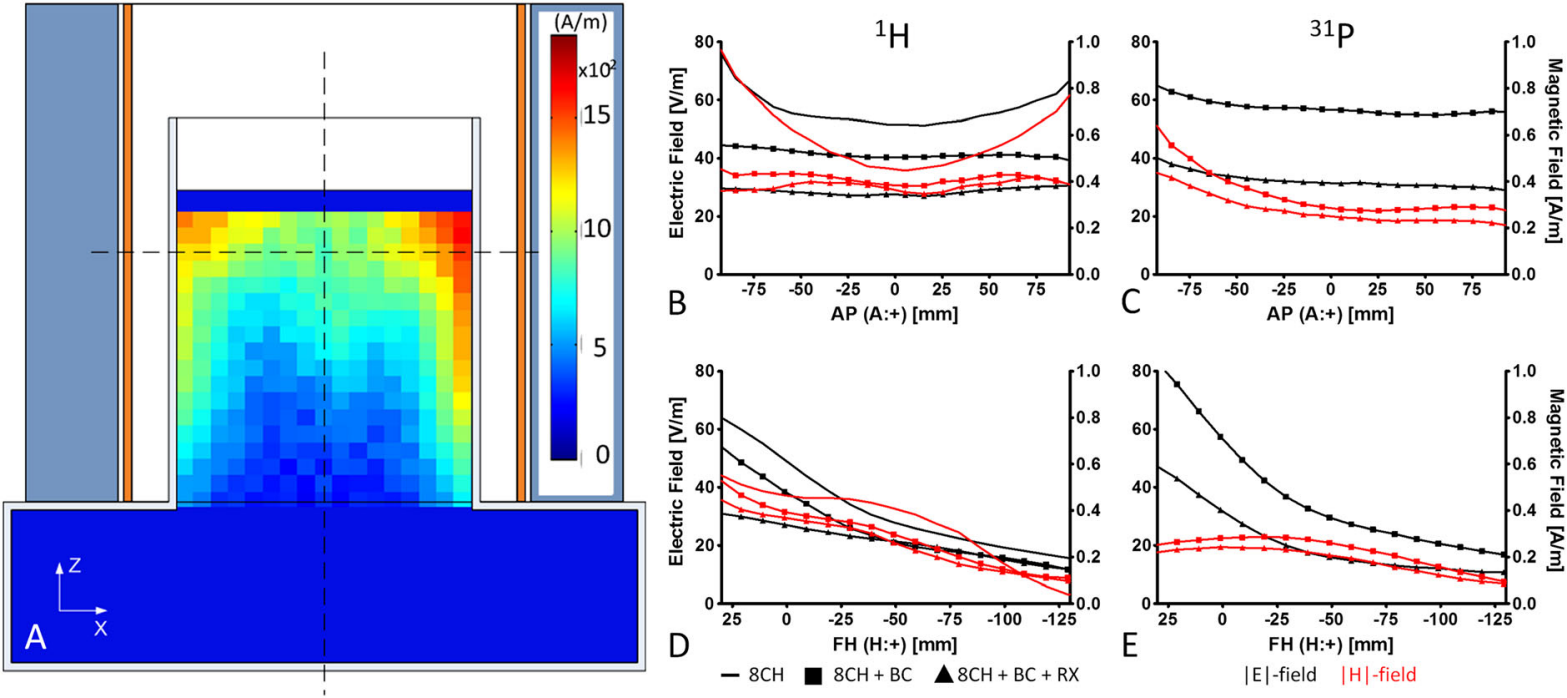
Results of the validation measurements using the field probes. (A) Two‐dimensional visualization of the coil setup surrounding the head–shoulder phantom as it was used to determine the magnitude of the |*E*| and |*H*| fields with the field probes. Included is a map of the proton |*H*| field as it was measured with the 8‐CH head coil only. The phantom was filled with tissue‐simulating fluid, having a conductivity of 0.98 S/m for ^1^H and 0.78 S/m for ^31^P, and a permittivity of 56.3 for ^1^H and 76.5 for ^31^P. (B–E) Profiles for each individual |*E*|‐ and |*H*|‐field measurement (these profiles were taken along the dashed lines (A)) with (B, D) ^1^H and (C, E) ^31^P. These measurements were made (i) for the 8‐CH head coil only, (ii) for the 8‐CH head coil with ^31^P BC inserted and (iii) for the complete setup with receive coil. These measurements were corrected for input power and hence are directly comparable to those of Reference 25. Note that |*E*| and |*H*| fields decrease when more components are present.

Simulations (CST Studio Suite, CST AG, Darmstadt, Germany) of the setup were made to assess homogeneity of the ^1^H‐field and to investigate couplings between the two volume coils. Those simulations were performed with and without the BC inserted in the 8‐CH head coil. The receive array was not used in the simulations, but validated with bench measurements. We assumed that existing SAR simulations for the 8‐CH head coil could be maintained when field homogeneity was not altered by the insert 19, 25. We validated these simulations, and the possible additional influence of the ^31^P Rx array, by assessing the |*H*|‐ and |*E*|‐field distributions at both resonances in a phantom filled with head tissue simulating liquid (^31^P, *ε*_r_ = 76.5, *σ* = 0.78 S/m; ^1^H, *ε*_r_ = 56.3, *σ* = 0.98 S/m). Field probes (Schmid & Partner Engineering AG (SPEAG), Zürich, Switzerland, probes H3DV7 & ES3DV2) were used to collect the maps in a sagittal (AP × FH 190 × 190 mm^2^) and coronal (LR × FH 160 × 190 mm^2^) plane in the phantom. Data were collected every 10 mm while probing through the complete coil setup with and without the ^31^P Rx array insert at the desired frequency (1 W). Both planes were positioned along the midline of the phantom (Fig. 3A).

### ^31^P MR of phantom and volunteers

The complete functionality of the coil setup was checked with the following tests: (i) 90° flip angle calibration of the BC, (ii) determination of the SNR increase of the ^31^P signal when acquiring data with the Rx array instead of the BC, (iii) obtaining the noise correlation matrix of the 7‐CH Rx array, and (iv) ^1^H *B*_1_^+^ shimming with all ^31^P components present. For these tests a cylindrical phantom (diameter 16 cm and volume ~ 5 L) filled with inorganic phosphate (P_i_) (30 mM) (and 2% agar) was used. For an additional *in vivo* test two healthy volunteers (males, 24 years and 26 years) were scanned after giving written informed consent.

A repeated slice selective pulse acquire sequence (6 ms excitation pulse (sinc), *T*_R_ = 15 s, single shot) selecting a 40 mm thick axial slice through the center of the coil was used for flip angle calibration, where the RF amplitude of the excitation pulse was varied to find the maximum amplitude of the peak present in the phantom (P_i_), which approximates a 90° excitation.

Two 3D MRSI data sets of the same cylindrical phantom were obtained to determine the SNR performance of the ^31^P coils. In both experiments, we excited the ^31^P signal using the BC, but acquired the signal with the BC in one, and acquired the signals with the ^31^P Rx array in the other experiment. Signals from the individual receive channels were combined using the WSVD combination method 8, comprising (i) a very short noise prescan that is used to decorrelate the single‐element spectra (or FIDs) and (ii) computation of the maximum likelihood combined spectrum. The Rx array was not present in the first test with the BC alone, thus before the second test the phantom was accurately repositioned after the Rx array was inserted. The 3D pulse–acquire MRSI experiment had the following parameters: *T*_R_/acquisition delay 1000/0.1 ms, flip angle 40°, hard pulse duration 0.3 ms, field of view (FOV) 240 × 240 × 200 mm^3^, matrix 12 × 12 × 8, elliptical *k*‐space acquisition with 100% Hamming filter, vector size 1024 and *T*_acq_ 5 min 15 s. The spectra from the MRSI data were fitted with Metabolite Report, a work in progress package from Siemens Healthcare (Erlangen, Germany). Metabolite Report performs automated, prior‐knowledge‐based, complex fitting in the time domain and has previously been applied to 7 T ^31^P spectroscopy data of the prostate 13. Images of the fitted P_i_ signal were created by interpolating its initial 12 × 12 × 8 matrix to a 256 × 256 × 8 matrix. They were then convoluted by a disk‐shaped kernel and masked. These images were then used to determine the local signal gain when using the Rx array as compared with the BC. An additional examination without RF excitation pulses was performed to obtain the noise correlation matrix.

In an *in vivo* study we investigated the ability to use *B*_1_ shimming and the ability to acquire 3D ^31^P MRSI in the human brain with and without NOE enhancement on a single volunteer (24 years) (*T*_R_/acquisition delay 1500/0.10 ms, flip angle 45°, matrix 12 × 12 × 8, FOV 240 × 240 × 240 mm^3^, pulse length 0.3 ms, elliptical *k*‐space acquisition with 100% Hamming filter, *T*_acq_ = 7 min 48 s). A high‐resolution 3D ^31^P MRSI using NOE enhancement with an approximated true voxel size of 3.0 cm^3^ was acquired from the second volunteer (26 years). This time data was collected with the Rx array, providing a reduced FOV. Parameters used for this sequence were *T*_R_/acquisition delay 500/0.10 ms, flip angle 30°, FOV 140 × 140 × 100 mm^3^, matrix 14 × 14 × 10, NSA = 6 and *T*_acq_ 15 min 2 s; all other parameters were kept the same. As the *T*_R_ is reduced and we want the maximum signal per unit time, we reduced the flip angle to the Ernst angle for PCr (30°) with *T*_1_ = 3.4 ± 0.3 s 14. In each examination the NOE enhancement was generated by saturating the water signals (*γB*_1_ = 30 Hz) using the wideband alternating‐phase low‐power technique for zero residual splitting (WALTZ‐4, technique originally intended for decoupling) 26 during the full *T*_R_, except during the 204 ms of signal acquisition. Other examinations within the protocol were the same for both volunteers. After *B*_0_ and *B*_1_ shimming the whole brain, a 3D *T*_1_‐weighted image was acquired with an MPRAGE (magnetization prepared rapid acquisition gradient echo) pulse sequence. For this sequence the following parameters were used: *T*_R_/*T*_I_/*T*_E_ 2500/1100/1.270 ms, resolution 1 mm^3^, *T*_acq_ 3 min 58 s. Then we calibrated the flip angle for ^31^P, and we optimized the *B*_1_ shim for the occipital lobe.

## Results

### Bench tests

#### RF coils

The ratio of unloaded‐to‐loaded quality factors (*Q*_U_/*Q*_L_) for the BC was 110/30 = 3.7. For the receive array it was (155 ± 20)/(63 ± 5) = 2.7 ± 0.3, with a range of 124–181 for *Q*_U_ and 57–69 for *Q*_L_.

#### Simulations and RF field measurements

Simulations were used to determine the influence of the ^31^P BC at the ^1^H field when it is inserted into the 8‐CH head coil. These showed that standard 12.5 mm wide copper tape for the legs created a signal void a few centimeters inside the phantom: coinciding with this void was a phase singularity (Fig. 2B, F). Simulations showed that the signal void and the phase singularity could be removed when the width of the legs was reduced to 4.5 mm (Fig. 2C, G), although with reduced sensitivity. The sensitivity increased and could be related to the field intensity of the 8‐CH head‐coil without BC when the tank circuitry was added to the BC (Fig. 2H).

The field probe measurements visualized the field distribution for both nuclei. For the ^1^H field in general, we concluded that the overall field distribution in the phantom was comparable to that from the reference coil 25, but that amplitudes in the center had been decreased for this new setup. This was shown by the coronal map of the |*H*| field at 297 MHz for the 8‐CH head coil only (Fig. 3A). Note that the 8‐CH head coil was tuned and matched to the resonance frequency with the BC inserted.

The proton |*H*|‐ and |*E*|‐field amplitudes at both resonances for the coil assembly in different configurations (Fig. 3) illustrated that the amplitudes of both field types decreased with the BC inserted. Signals did not decrease further with the Rx coil present. Moreover, the stronger decrease in |*E*| field (causing SAR deposition) compared with the |*H*| field confirmed that it was safe to use this coil assembly with the original 8‐CH safety margins.

Comparing these results with the 8‐CH proton setup without any additional coils 19, 25 enabled us to use the same maximal permissible input power, since the magnitude of the electric field is reduced after insertion of the additional components, providing an even greater safety margin in the case of the new ^1^H coil assembly. For ^31^P the maximum permissible input power was calculated based on a worst‐case approximation (losses in the feeding network and RF coil were neglected) for the head‐averaged SAR according to the rationale given in the IEC 60601‐2‐33 for volume transmit coils: *P*_max_^31P^ = SAR_head,limit_ *m*_head_.

### ^31^P MR of phantom

Calibration of the RF power to reach a 90° flip angle for the ^31^P signals in a phantom as well as in the human brain showed that the maximum signal intensity coincided with a reference *B*_1_^+^ amplitude of 29 μT. This resulted in a maximum achievable *B*_1_^+^ amplitude for ^31^P of approximately 56 μT for this coil setup (on average this value was a little lower, 45–50 μT).

**Figure 4:**
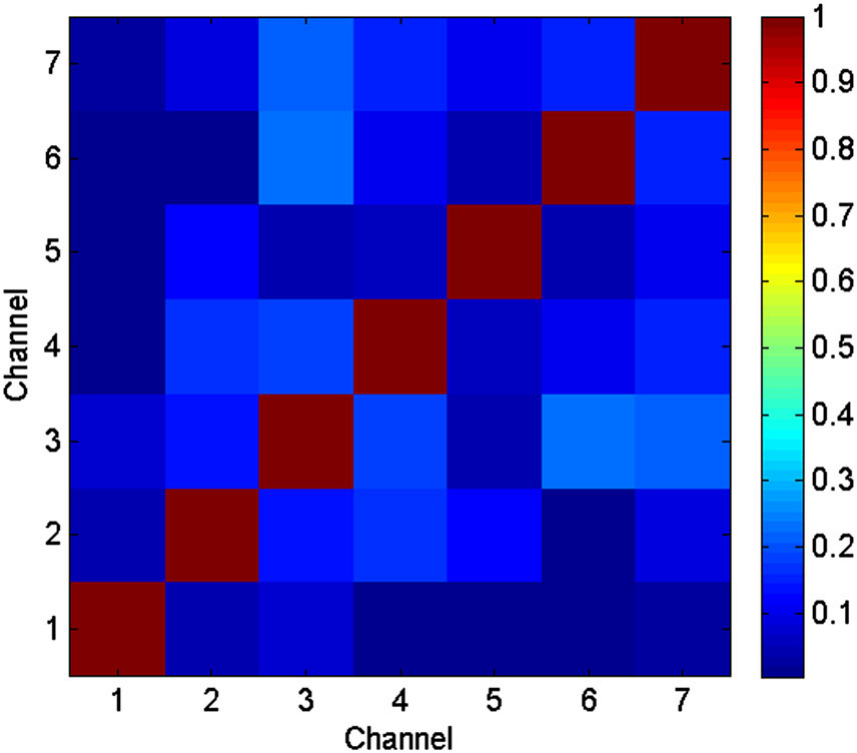
Noise correlation matrix of the 7‐CH Rx array. All channels are properly decoupled, because noise does not correlate significantly. The average noise correlation over all elements was 10 ± 7%, with its maximum of 23% found between elements 3 and 6.

The noise correlation matrix (Fig. 4) confirmed that coupling between all elements was minimized as a result of overlap and pre‐amplifier decoupling. The average noise correlation over all elements was 10 ± 7%, with its maximum of 23% found between elements 3 & 6.

**Figure 5:**
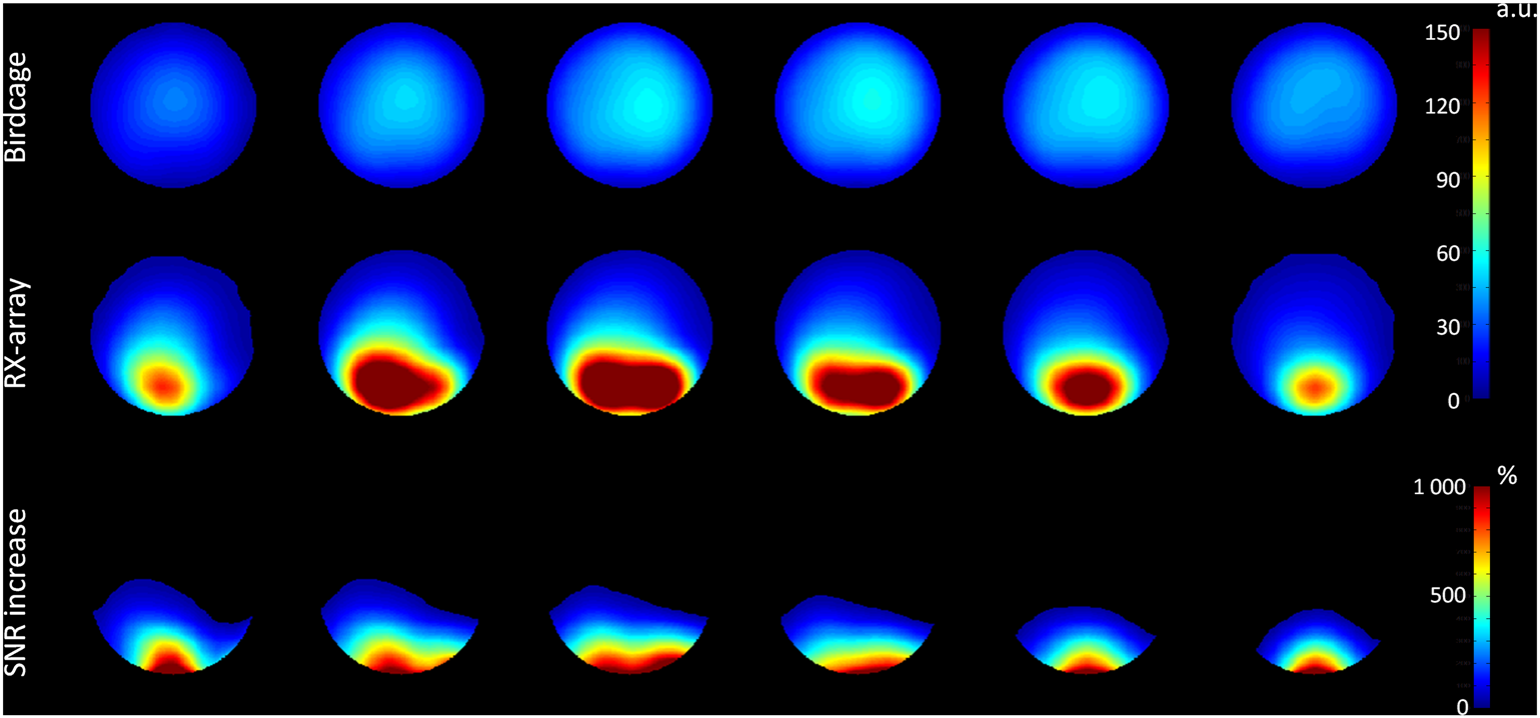
Interpolated SNR images of a spherical phantom containing 30 mM inorganic phosphate. The images were obtained with the BC coil (top row) and with the local Rx array (middle row). The gain in SNR is more than sevenfold close to the receive array. Also note the uniform *B*_1_ field when data was solely acquired with the BC.

As expected, the SNR increased substantially when the Rx array was used for signal acquisition as compared with the BC. An increase in SNR was visible up to 7 cm inside the phantom, with a sevenfold increase up to 2 cm inside the phantom (Fig. 5).

### ^31^P MR of volunteers

**Figure 6:**
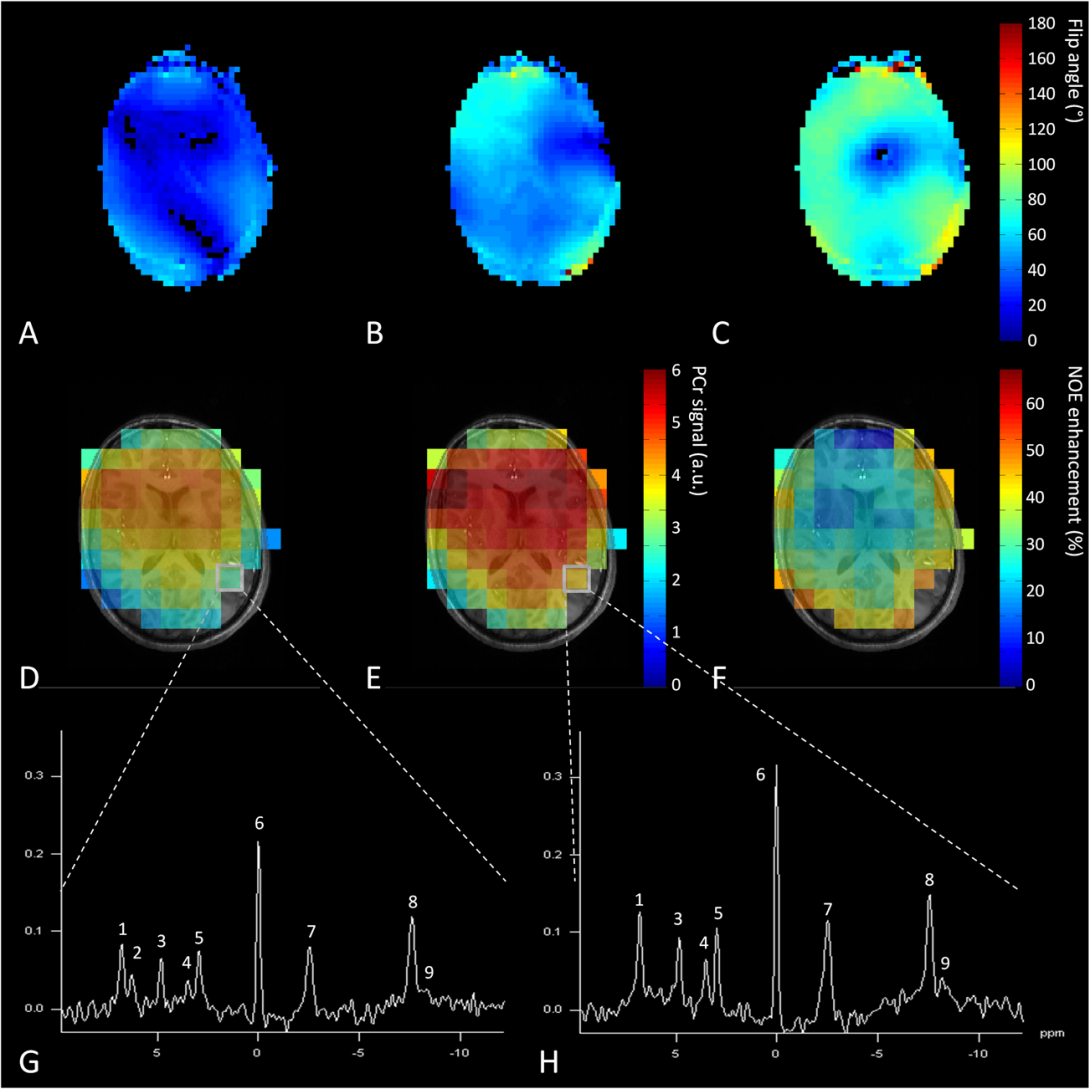
(A–C) Results of *in vivo* experiment using the complete setup showing different *B*_1_^+^ maps of the ^1^H field: (A) in CP− mode, (B) homogenized for the complete brain, and (C) locally optimized for occipital lobe. Showing the ability to use *B*_1_ shimming with the coil setup. Background images were acquired with the 8‐CH ^1^H head coil driven with global *B*_1_ optimization, showing fairly homogeneous field distribution. (D–F) ^31^P spins were excited and signal was acquired with the BC (D) without and (E) with NOE enhancement of PCr; (F) the global enhancement map. (G, H) An example of ^31^P spectra taken from the same voxel (G) without NOE enhancement and (H) with NOE enhancement. Spectra were obtained in 7 min 48 s with an approximate voxel size of 38 cm^3^. The metabolites present in these spectra are (1) phosphoethanolamine (PE), (2) phosphocholine (PC), (3) P_i_, (4) glycerophosphoethanolamine (GPE), (5) glycerophosphocholine (GPC), (6) PCr, (7) γ‐ATP, (8) α‐ATP and (9) NADH.

The *in vivo* experiments were successful. We could obtain detailed *T*_1_‐weighted images with the 8‐CH ^1^H head coil driven in CP+ mode (Fig. 6, background) and were able to shim the ^1^H *B*_1_^+^ field (Fig. 6A–C). This allowed us to maximally enhance ^31^P signals using a *B*_1_‐shimmed setting for specified regions. For the whole brain, we plotted the metabolite map of PCr when measured with and also without NOE enhancement (Fig. 6D, E). An enhancement map showed the signal increase, expressed as a percentage (Fig. 6F); the main features of this enhancement map coincided with the ^1^H *B*_1_^+^ field (Fig. 6C). The overall enhancement of the PCr signal was 30 ± 9%.

**Figure 7:**
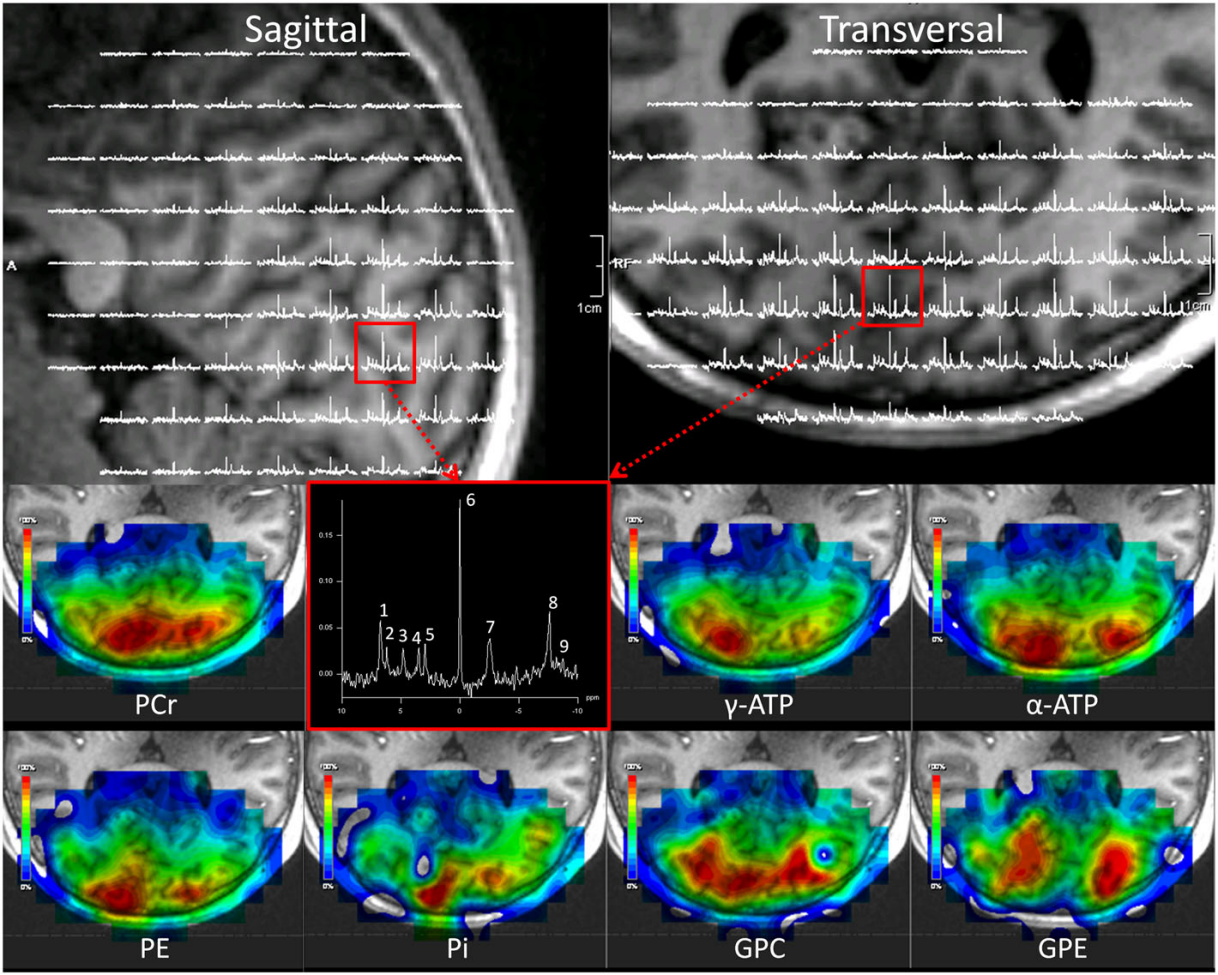
High‐resolution 3D MRSI using all available options to increase SNR. The signals were enhanced using NOE and were received with the local receive array. Note the exquisite quality of the spectra. Top left, a sagittal view of the human brain overlapped with ^31^P spectra as acquired with the receive array. Top right, a transversal view of the human brain also overlapped with a spectral map covering the occipital lobe. Bottom two rows, metabolite maps of seven different ^31^P compounds. The colors show the distribution of the metabolites (scaled 0–100%) represented by the fitted integral. Note that in only two voxels were low‐SNR metabolites (P_i_ and GPC) fitted incorrectly within the FOV of the array coil. Moreover, note the difference in spatial distributions of the different metabolites, which arise not only from the *B*_1_‐receiving profile (anterior–posterior), but also from differences between gray and white matter (left–right). A spectrum with high quality (3 cm^3^ voxel), as acquired in 15 min in the occipital lobe (red box), is shown too. Not shown in this spectrum is the β‐ATP, as the bandwidth of the pulse to properly excite this metabolite was insufficient. (1) PE, (2) PC, (3) P_i_, (4) GPE, (5) GPC, (6) PCr, (7) γ‐ATP, (8) α‐ATP and (9) NADH.

When acquiring signals with the local receive array, we could reduce the FOV and hence increase spatial resolution. Combined with local NOE enhancement and an optimized signal combination method (i.e. WSVD combination), we obtained ^31^P spectra from relatively small voxels (3.0 cm^3^) in 15 min, with narrow linewidths for PCr (9.6 ± 1.9 Hz) and high SNR (17.8 ± 3.7) (Fig. 7), up to 5 cm into the occipital lobe.

The SAR for the experiments with NOE enhancement was evaluated using the real‐time SAR monitoring system 27. The time‐averaged total input power for these experiments (not incorporating coil losses) was 10.9 W for ^1^H and 2.2 W for ^31^P.

## Discussion

In this work, we have described the design, construction, and safety validation of a dedicated coil setup for 7 T containing three main components: an 8‐CH multi‐transmit ^1^H head coil, an insertable and actively tunable BC coil for ^31^P and a local 7‐CH ^31^P Rx array. The setup featured multi‐transmit capabilities on the ^1^H channels, volume excitation for ^31^P, and a large local SNR improvement close to the receive array. This increase in sensitivity in combination with the limited FOV of local receive coils enabled ^31^P MRSI at higher spatial resolution, and may be used to reduce scan time.

The tuning and matching of the 8‐CH ^1^H head coil had to be adapted, because its initial tuning frequency dropped below the resonance frequency of the system. Therefore we tuned it to the correct frequency and adapted the matching to become 50 Ω (loaded) with the BC present. Despite this adaption, we noticed a reduction in sensitivity of the ^1^H signal, which is probably caused by the large quantity of conductive material present in the BC itself. Although this might block part of the signal, as reported for high‐density receive arrays 28, we did not observe a change in the shape of the field distributions in the phantom measurements.

Although not shown here, it is worth noting that the homogeneity of the BC was hardly altered by the addition of the local Rx array. Each element in the receive array was actively detuned, meaning that a current was used to forward bias a parallel positioned PIN‐diode, bringing the element off resonance during BC transmit. We added no additional safety features, such as a second trap with passive diodes 29 or an RF fuse, to the individual loops in order to avoid a possible decrease in SNR. Instead, we considered (and verified) that, should a parallel‐positioned PIN‐diode break down, a short circuit would be created and hence the coil element would be detuned, which makes it intrinsically safe. However, this feature does not detect a moment when no current flows through the PIN‐diode (e.g. when a cable is disconnected). Therefore, we added a current sensing circuit inside the interface, which activates and stops the scanner immediately if no current goes to the PIN‐diodes.

The SNR increase by exciting the spins via the BC and receiving their signal with the array coil may have been biased, as the BC for reception may have had degraded performance. The PIN‐diodes for tuning the BC were positioned in series within the legs: when forward biased (tuned) they have a low additional series resistance; when the PIN‐diodes are reverse biased (detuned) this resistance will increase significantly. However, the coil has a relatively high *Q*_U_ (110), and when the *Q*_U_/*Q*_L_ ratio is above 3.5 tissue loading dominates over coil losses. Hence, the introduction of PIN‐diodes in the BC did not degrade its performance in the first place.

Increasing SNR by separating Rx and Tx surface coils for ^31^P spectroscopy has been reported before 30, 31, where Avdievich and Hetherington were the first to design and develop a homogeneous transmit volume coil 5, which was the basis for our work. Their coil was designed as an actively detunable double‐tuned transverse electromagnetic (TEM) head coil for 4 T. The two setups have similar properties and both can provide anatomical reference images and ^31^P MRSI of the patient without removal of the coil or the patient. The advantage of our design over theirs is the ability to use *B*_1_ shimming. Moreover, the size of the individual elements of the Rx ^31^P array in our design was smaller, resulting in a local increase in SNR.

It was shown by Lei *et al*. that signal from PCr in the human brain can be increased by 24.3 ± 1.6% using NOE 14. However, this effect depends on the amplitude and distribution of the ^1^H *B*_1_ field (Fig. 6C, F) and can therefore vary spatially 13. This principle can be exploited to enhance specific regions. If NOE is used, it is essential to reach a threshold ^1^H *B*_1_^+^ value in a certain area to achieve maximum NOE enhancement. An additional ^1^H *B*_1_^+^ map is therefore needed to assess the locations where the threshold *B*_1_^+^ was reached. ^31^P *B*_1_^+^ does not affect the NOE variation. With the current coil setup, an overall enhancement of 30 ± 9% for the PCr signal could be reached in the human brain. This higher enhancement compared with the data of Lei *et al*. may be related to the proton irradiation power of this coil and our choice of the WALTZ‐4 decoupling strategy.

Although the amplitude of the ^1^H signals was reduced with the ^31^P BC inserted, we were able to optimize the *B*_1_^+^ field and use this to enhance signals. Moreover, the irradiation of the water spins with low RF power led to a time‐averaged maximum input power of 10.9 W. Assuming the weight of an average head to be approximately 5 kg, this leads to a worst‐case head‐averaged SAR of 2.18 W/kg, which is already well below the IEC‐defined limit of 3.2 W/kg for the head. Within this approximation losses inside the coil (e.g. feed network, coil elements, etc.) are also present, thus the head‐averaged SAR would be reduced even further. Furthermore, when using phased array coils for exciting the MR signal instead of volume resonators, SAR deposition can be reduced significantly (up to 50%) while optimizing the *B*_1_^+^ distribution, as has been shown by van den Bergen *et al*. in the pelvic area 32. Although this was investigated at 3 and 7 T with a TEM coil, it was shown by Wang *et al*. that SAR levels are lower in microstrip coil designs at 7 T 33. These findings support the usage of this 8‐CH multiple‐array antenna. Moreover, the meander structures in the element decrease intrinsic coupling of the elements, and reduce the magnitude of the electric field at the ends of the elements 20. A striking fact from the same study is that SAR levels for BC coils, such as our new design for ^31^P, were higher at 7 T. Note that we optimized this BC coil for a lower frequency (120.3 MHz), and when using volume coils at these frequencies the head‐averaged SAR is usually the most critical aspect.

Most studies on ^31^P in the human brain at 7 T have been using surface coils to excite and receive the MR signal 14, 34, 35, except for that by Zhu *et al*. 36, who used a CP dual‐frequency volume RF coil. Although surface coils have a higher SNR, their *B*_1_ fields are inhomogeneous, hence the flip angle becomes position dependent should one use a hard pulse for excitation. By using adiabatic pulses these limitations can be circumvented, but at a cost of markedly increased pulse duration and concomitant increase in SAR deposition. Using the homogenous volume resonator present here allowed us to avoid SAR demanding adiabatic pulses, but rather use short (0.3 ms) hard pulses that have sufficient bandwidth to excite the spectrum (except β‐ATP). Note that Zhu *et al*. were not able to use these in their study 36.

In conclusion, the developed coil setup can be used to excite ^1^H and ^31^P signals at an ultra‐high field strength of 7 T. ^31^P signals can be acquired with an increased SNR by exploiting NOE enhancement and by receiving the signals with a local receive array.
